# Hair Cortisol Concentration, Weight Loss Maintenance and Body Weight Variability: A Prospective Study Based on Data From the European NoHoW Trial

**DOI:** 10.3389/fendo.2021.655197

**Published:** 2021-09-29

**Authors:** Sofus C. Larsen, Jake Turicchi, Gitte L. Christensen, Charlotte S. Larsen, Niklas R. Jørgensen, Marie-Louise K. Mikkelsen, Graham Horgan, Ruairi O’Driscoll, Joanna Michalowska, Cristiana Duarte, Sarah E. Scott, Inês Santos, Jorge Encantado, Antonio L. Palmeira, R. James Stubbs, Berit L. Heitmann

**Affiliations:** ^1^ Research Unit for Dietary Studies, The Parker Institute, Bispebjerg and Frederiksberg Hospital, The Capital Region, Frederiksberg, Denmark; ^2^ School of Psychology, Faculty of Medicine and Health, University of Leeds, Leeds, United Kingdom; ^3^ Department of Clinical Biochemistry, Rigshospitalet, Glostrup, Denmark; ^4^ Department of Clinical Medicine, Faculty of Health and Medical Sciences, University of Copenhagen, Copenhagen, Denmark; ^5^ Biomathematics and Statistics Scotland, Aberdeen, United Kingdom; ^6^ Department of Treatment of Obesity, Metabolic Disorders and Clinical Dietetics, Poznan University of Medical Sciences, Poznan, Poland; ^7^ Laboratório de Nutrição, Faculdade de Medicina, Universidade de Lisboa, Lisbon, Portugal; ^8^ Centro Interdisciplinar para o Estudo da Performance Humana, Faculdade de Motricidade Humana, Universidade de Lisboa, Lisbon, Portugal; ^9^ Centro de Investigação em Desporto, Educação Física, Exercício e Saúde (CIDEFES), Universidade Lusófona, Lisbon, Portugal; ^10^ Applied Psychology Research Center Capabilities & Inclusion (APPsyCI), Instituto Superior de Psicologia Aplicada (ISPA) - Instituto Universitário, Lisbon, Portugal; ^11^ The Boden Institute of Obesity, Nutrition, Exercise & Eating Disorders, The University of Sydney, Sydney, NSW, Australia; ^12^ Department of Public Health, Section for General Practice, University of Copenhagen, Copenhagen, Denmark

**Keywords:** hair cortisol, obesity, stress, weight loss, weight loss maintenance

## Abstract

Several cross-sectional studies have shown hair cortisol concentration to be associated with adiposity, but the relationship between hair cortisol concentration and longitudinal changes in measures of adiposity are largely unknown. We included 786 adults from the NoHoW trial, who had achieved a successful weight loss of ≥5% and had a body mass index of ≥25 kg/m^2^ prior to losing weight. Hair cortisol concentration (pg/mg hair) was measured at baseline and after 12 months. Body weight and body fat percentage were measured at baseline, 6-month, 12-month and 18-month visits. Participants weighed themselves at home ≥2 weekly using a Wi-Fi scale for the 18-month study duration, from which body weight variability was estimated using linear and non-linear approaches. Regression models were conducted to examine log hair cortisol concentration and change in log hair cortisol concentration as predictors of changes in body weight, change in body fat percentage and body weight variability. After adjustment for lifestyle and demographic factors, no associations between baseline log hair cortisol concentration and outcome measures were observed. Similar results were seen when analysing the association between 12-month concurrent development in log hair cortisol concentration and outcomes. However, an initial 12-month increase in log hair cortisol concentration was associated with a higher subsequent body weight variability between month 12 and 18, based on deviations from a nonlinear trend (β: 0.02% per unit increase in log hair cortisol concentration [95% CI: 0.00, 0.04]; *P*=0.016). Our data suggest that an association between hair cortisol concentration and subsequent change in body weight or body fat percentage is absent or marginal, but that an increase in hair cortisol concentration during a 12-month weight loss maintenance effort may predict a slightly higher subsequent 6-months body weight variability.

**Clinical Trial Registration:**

ISRCTN registry, identifier ISRCTN88405328.

## Introduction

Worldwide, obesity is one of the leading preventable causes of both morbidity and mortality (1–3). While existing approaches are generally effective for initial weight loss (WL), behavioural interventions have shown quite limited effects on long-term weight loss maintenance (WLM) (4). Thus, understanding the potential causes or predictors of WLM and weight regain can have important therapeutic implications. Dietary relapse and/or a reduction in physical activity are the obvious courses of weight regain, but there are likely numerous genetic, behavioural, metabolic and hormonal factors involved (5–8).

While the underlying mechanisms are not fully understood, a link between perceived stress and obesity has been suggested for decades (9). It has also been reported that perceived stress predicts weight regain after clinically significant WL (10). Several factors and pathways could potentially explain this relationship, including that psychological stress can affect the hypothalamic-pituitary-adrenal (HPA) axis and lead to an increase in cortisol secretion (11). This increase in cortisol may promote weight regain through an increase in appetite (8), but it has also been suggested that cortisol can directly promote fat disposition, as seen in Cushing’s disease where frequent symptoms are weight gain, increased body fat (BF) and central obesity (12).

In addition to an association between perceived stress and weight regain, some studies have shown tendency towards irregular eating patterns among individuals reporting higher levels of stress, an eating behaviour referred to as ‘stress eating’ (13–15). This could theoretically lead to a higher degree of fluctuation in caloric intake and thereby promote a higher body weight variability (BWV), which by itself has been suggested as a predictor of future weight gain (16–18). However, to the best of our knowledge, an association between stress and BWV has neither been explored using self-reported nor biological measures of stress.

Measurement of cortisol in blood, saliva or urine are often used as biological markers of stress. However, an important limitation to these measures is that they provide information on current cortisol level, and consequently reflects acute, rather than chronic, stress. In contrast to these measures, hair cortisol concentration (HCC) is a biomarker for assessing chronic stress, by reflecting cortisol exposure during the period of hair growth (19). While previous studies have shown inconsistent associations between HCC and perceived stress (20), the measure has been shown to align with saliva cortisol production measured continuously over a corresponding one-month period (19).

Several studies have shown HCC to be associated with adiposity at a cross-sectional level (21–24), but the relationship between HCC and long-term WLM, or longitudinal weight change in general, is largely unknown and as a consequence the direction of causality has not yet been determined.

The present study is an ancillary study based on data from the European NoHoW trial, where the aim was to test the efficacy of an evidence-based digital toolkit, targeting self-regulation, motivation, and emotion regulation, on WLM among British, Danish, and Portuguese adults (25). Using the NoHoW data, the aim of the present study was to examine the longitudinal associations between HCC, WLM and body weight variability. More specifically, we examined: 1) the association between baseline HCC and subsequent changes in body weight (BW) (primary outcome), change in BF%, and BWV (secondary outcomes) during 6, 12 and 18 months of follow-up, 2) the association between 12-month change in HCC and 12-month develop in outcome measures during the same period, and 3) the association between initial 12-month change HCC and subsequent development in outcome measures between month 12 and month 18.

## Materials and Methods

### Study Population

A detailed description of NoHoW can be found elsewhere (25). The trial was registered with the ISRCTN registry (ISRCTN88405328). NoHoW was a randomised controlled trial testing the efficacy of an information and communications technology (ICT)-based toolkit to support WLM in the United Kingdom (Leeds), Denmark (Copenhagen), and Portugal (Lisbon). At baseline examinations between March 2017 and March 2018, all participants were randomly allocated to one of four arms: (1) self-monitoring only (self-weighing and activity tracker), (2) self-regulation plus motivation, (3) emotion regulation, or (4) combined self-regulation, motivation, and emotion regulation. Participants were followed during a 12-month period for change in BW (primary trial outcome), body composition, biomarkers, dietary intake, physical activity, sleep, and psychological mediators/moderators of WLM (secondary trial outcomes). In addition to the primary follow-up visit at month 12, measurements were collected at month 6 and month 18. Participants were 18 years or older, had achieved a verified and clinically significant WL of ≥5% within the 12 months prior to inclusion, and had a BMI of ≥25 kg/m^2^ before losing weight. The exclusion criteria were as follows: achieved WL due to illness or surgical procedures; pregnancy or breastfeeding; involvement in other research intervention studies that confound with the aims of the intervention; inability to follow written material or telephone conversations in the English, Danish, or Portuguese language (depending on the trial centre); diagnosis of an eating disorder; diagnosis of any condition that may interfere with increasing mild to moderate physical activities and that is unstable (i.e., untreated or unable to be controlled by medication); recent diagnosis of type 1 diabetes; extensive travel plans (e.g., more than four weeks); or living in the same household as existing participant in the trial (25). A total of 1,627 participants were enrolled in the NoHoW trial.

The present ancillary study was based on information collected at baseline, 6, 12 and 18-month follow-up visits. We further excluded participants with missing information on baseline HCC (n=496), no follow-up information on the primary outcome (BW) (n=157), and missing information on selected covariates (n=188). A total of 786 participants had information on HCC, selected covariates and information on BW from at least one follow-up visit (month 6 [n=773], month 12 [n=713] and month 18 [n=668]). A slightly lower sample size was used for the secondary outcomes (BF% and BWV). A flowchart showing the selection of participants can be found in **Supplementary Figure 1**.

### Ethics

The study was conducted in accordance with the Helsinki Declaration. Ethical approval was granted by local institutional ethics committees at the Universities of Leeds (17-0082; 27 February 2017), Lisbon (17/2016; 20 February 2017) and the Capital Region of Denmark (H-16030495; 8 March 2017).

### Measurements

#### Hair Cortisol Concentration

Hair samples were cut from the posterior vertex as close to the scalp as possible. Between 10-30 mg of the hair from 2 cm closest to the scalp was cut in small pieces and dissolved in 1 mL methanol and incubated at ultrasound sonication for 30 minutes, followed by 18 hours at 52°C in a shaking incubator (300 rpm.). Hair samples were not collected among participants with less than 2 cm of hair. The methanol was transferred to a new tube and evaporated to dryness under a stream of nitrogen at 45°C. Dried samples were stored at -20°C until analysis. Before analysis, samples were re-dissolved in 500µl PBS pH 8 and centrifuged at 2000 rpm for 2 min. Reconstituted samples were analysed with a cortisol ELISA assay (Alpco.com). The measurement range of the assay was 0.15-50 ng/mL. We used three levels of control specimens to show the inter-assay variation as the inter-assay precision might be different depending on the measurements level. The inter-assay variation was 16.7% (mean 0,22 ng/mL), 11,4% (mean 9,8 ng/mL) and 7.7% (mean 49.2 ng/mL). HCC was expressed as pg cortisol/mg hair.

We aimed to analyse 10-30 mg of hair. However, especially among the male participants, the collected samples did not consistently contain enough hair to meet this criterion. Thus, as we found a direct association between weight of the hair sample and HCC, weight of hair samples (mg) was included in sensitivity analyses.”

#### Body Weight and Body Fat Percentage

At baseline and follow-up visits, BW was measured to the nearest 0.1 kg and height was measured at baseline to the nearest 0.1 cm using the Seca 704s (SECA, Hamburg, Germany) combined stadiometer and electronic scale. Body composition was estimated from bioelectrical impedance using the ImpediMed SFB7 device (ImpediMed, Inc, Sydney, Australia) (software version 5.4.0), following the manufacturer’s instructions. We used the Moissl BMI modification of the mixture theory equations to determine BF%, a method that has been found appropriate over a wide range of body compositions (26). Changes in BW and BF% between baseline and 6, 12 or 18 months and between month 12 and 18 (follow-up values minus baseline values) were included in analyses as continuous variables.

#### Body Weight Variability

In addition to the weight measurements conducted at the official centre visits, all participants were provided with a Fitbit Aria smart scale (Fitbit Inc, San Francisco, CA, USA). This scale was linked to a personalised Fitbit account and the data were retrieved *via* a Fitbit application programming interface (API) to an online data hub. The Fitbit Aria scale has been shown to have excellent agreement with a calibrated research grade SECA 769 scale (27). All participants were instructed to weigh themselves on the Fitbit Aria scale at least twice per week for the duration of the study. From this, BWV was estimated from the Root Mean Square Error (RMSE) (28–30). Moreover, we included an additional measure, which we have previously developed to overcome limitations of commonly used approaches (including assumption of linearity in weight trajectory), termed the non-linear mean deviation (NLMD) method. Detailed information on the methods used to calculate BWV has been published elsewhere (30, 31). In brief, RMSE was estimated by taking the mean square of the relative residual error in the linear relationship between BW and time. The NLMD was calculated by fitting a locally estimated scatterplot smoothing (LOESS) regression to the BW data which acts as a smoother. The fit of the regression was then subtracted from the observed BW and the relative mean deviation from the non-linear trend was calculated. RMSE and NLMD from baseline to month 6, 12 and 18 and between months 12 and 18 were included in the analyses as continuous variables (%).

#### Covariates

Participants provided verified information on WL during the 12 months prior to inclusion at baseline (by a health professional, WL counsellor/friend, WL programme record booklet, diary, smartphone app, or before/after photographs), and WL was included in the analyses as a continuous variable (kg). At baseline, all participants were allocated to an intervention arm and this information was included as a categorical variable: (1) self-monitoring only (self-weighing and activity tracker), (2) self-regulation plus motivation, (3) emotion regulation, or (4) combined self-regulation plus motivation and emotion regulation. Participants were asked to report their smoking status, and were categorised as current, previous or never smokers. Information was also collected on alcohol consumption, which was included using the following 6 categories: every day, 5–6 times a week, 3–4 times a week, twice a week, once a week, and less than once a week. Information on highest level of education was provided and categorised according to the International Standard Classification of Education (ISCED) (32) as high, medium, low, or other (including educations not classified by ISCED. Age (continuous variable) and sex was also included. Objectively measured physical activity was measured using the Fitbit Charge 2 (33, 34). The device was updated to the latest firmware and worn on the nondominant wrist of the participant. Participants were instructed to wear the Fitbit Charge 2 for the duration of the study, apart from during water activities (e.g., showering) and when charging the device. In the present study, we used 14 days of sleep data recorded from day 3 to day 16 of the intervention. Days 1 and 2 were excluded to ensure all devices had been properly set up. When no heart rate data were available from the Fitbit, we considered it as non-wear time. To avoid loss of data due to connectivity issues, gaps of less than 10 minutes were imputed with the average of the last measured and the next observed heart rate. Minute-level data were aggregated to hourly data and missing time was determined per hour. Total number of steps were divided by the number of minutes the device was worn, on the assumption that data missing within each hour were most representative of missing data. Hours with more than 30 minutes of missing data were removed from the data. Next, hourly averages were summed per day, a minimum of 21 valid hours were required for a valid day. Total steps were then averaged across the 14-day period if at least 6 valid days and 2 weekend days were available. From this, physical activity measured as average steps/day (continuous variable) was included. Lastly, information on centre/country of residence was included.

In sensitivity analyses, information on sleep habits (sleep duration and sleep onset variability) and psychosocial stress were added. Objectively measured sleep was also collected using the Fitbit Charge 2. Sleep duration and sleep onset was assessed across the 14 days close to baseline examinations using a previously described approach (35, 36). Mean daily sleep duration was calculated and included in analyses as a categorical variable: <6, 6–<7, 7–<8, 8–<9, and ≥9 hours. Moreover, mean sleep onset was assessed by identifying the beginning of each main sleep period in minutes from midnight (e.g., 23:00 = −60 minutes and 01:00 = 60 minutes). From this, variability in sleep onset was estimated for each individual using the standard deviation across all nights recorded and included as a continuous variable (hours). Psychosocial stress was assessed with the short version of the perceived stress scale (PSS) (37, 38). This scale has the following four items that focus on the assessment of stress and coping over the preceding month: ‘How often have you felt that you were unable to control the important things in your life?’ ‘How often have you felt confident about your ability to handle your personal problems?’ ‘How often have you felt that things were going your way?’ and ‘How often have you felt difficulties were piling up so high that you could not overcome them?’. Responses were made on a 5-point Likert scale (never, rarely, sometimes, often, and very often). The items were then summed to give a total perceived stress score with a range of 0 (least stressed) to 16 (most stressed), which was included as a continuous variable.

### Statistical Analyses

All statistical analyses were guided by an analysis plan developed prior to analysing the data (**Supplementary Text 1**). The sample size of the NoHoW trial was determined with the main purpose of having sufficient statistical power to detect a potential effect of the intervention on change in BW (25). As the present results represent an ancillary study, the sample size was not determined specifically for the purpose of these analyses. However, the pre-established sample size of 786 individuals available for the current study gave approximately 80% power to detect correlations of 0.1 or greater.

Characteristics of study participants were presented as medians and corresponding interquartile ranges (IQR) for continuous variables and as percentages for categorical variables. Between sex differences were tested using Wilcoxon rank-sum test or chi-squared test.

Due to the highly skewed nature of HCC, this variable was log-transformed to attain a normal distribution. For descriptive purposes, both actual values and log-transformed values are presented in descriptive part of the result section.

Linear regression analyses were conducted to assess the associations between baseline log HCC and subsequent 6, 12 and 18-month change in BW and BF%, in addition to BWV over the same time periods. Moreover, we examined the association between 12-month concurrent changes in HCC and the outcome measures, and the association between initial change in log HCC from baseline to month 12 and subsequent change in outcome measures between month 12 and month 18.

First, crude models, including information on outcome, exposure and baseline measure only were conducted. Secondly, adjusted analyses with added information on initial WL, smoking status, frequency of alcohol consumption, physical activity, education, age, intervention status (arm allocation), centre (country of residence) and sex as potential confounding factors were conducted.

Model assumptions (investigating linearity of effects on outcomes, consistency with a normal distribution, and variance homogeneity) were assessed through visual inspection of histograms and residual plots. Some models with BWV as the outcome measure showed slight deviation from the assumption of normally distributed residual. However, as sensitivity analyses with log-transformed outcomes gave essentially the same results, analyses with non-transformed outcome measures are presented throughout.

Sex and intervention interactions were tested in all analyses by adding product terms to the models, and subgroup analyses were conducted if significant interaction was observed.

All statistical tests were two-tailed with a significance level at 0.05. BWV estimates were calculated in R version 3.4. Analyses were performed using Stata SE 14 (StataCorp LP, College Station, Texas, USA).

#### Sensitivity Analyses

To get associations independently of self-reported stress, sensitivity analyses additionally adjusted for the PSS were conducted. Moreover, since stress is closely related to sleep (39), and we have previously shown an association between sleep onset variability and weight regain among the NoHoW participants (35), sensitivity analyses were conducted adjusting for sleep duration and sleep onset variability. Analyses adjusted for hair washing frequency and use of hair dye were conducted as well, although the evidence for potential influence on HCC from these factors is inconsistent (21, 40). Sensitivity analyses were also performed adjusting for weight of the hair sample, as we did not have 10-30 mg of hair for all individuals and we found that log HCC was positively correlated with weight of the hair samples (r=0.2). All analyses were also conducted excluding participants with less than 10 mg of hair at baseline (n=213) and follow-up (n=74). Finally, we performed sensitivity analyses excluding participants currently treated with any form of medication (n=301)

## Results

A total of 172 men and 614 women were included in the study. A higher median baseline age was observed among women [46.4 years (IQR: 38.2, 54.2)] than men [42.2 years (IQR: 36.2, 58.5); *P* < 0.001]. The median baseline BMI was 29.0 kg/m^2^ (IQR: 28.6, 29.1) with no difference between men and women (*P* = 0.321). The median weight change prior to baseline was -10.0 kg (IQR: -13.6, -7.0), and again with no difference between sexes (*P*=0.451) (**Table 1**).

**Table 1:** Baseline characteristics of men and women from the NoHoW study.

	All (n = 786)^1^	Men (n = 172)	Women (n = 614)	P^3^
Age (years)	45.2 (37.5, 53.2)	42.2 (36.2, 58.5)	46.4 (38.2, 54.2)	<0.001
Height (cm)	167.3 (162.8, 173.5)	177.5 (173.0, 181.6)	165.5 (161.5, 170.0)	<0.001
BMI (kg/m2)^2^	29.0 (25.9, 32.7)	28.6 (25.8, 31.7)	29.1 (25.9, 33.0)	0.321
Initial weight loss (kg)	-10.0 (-13.6, -7.0)	- 10.0 (-14.0, -7.0)	-10.0 (-13.5, -7.0)	0.451
Frequency of alcohol consumption (%)				
Every day	1.9	3.5	1.5	<0.001
5-6 times a week	3.4	5.2	2.9	
3-4 times a week	11.2	17.4	9.5	
Twice a week	15.3	20.4	13.8	
Once a week	10.4	14.0	9.5	
< once a week	57.8	39.5	62.9	
Smoking status (%)				
Current	7.5	11.1	6.5	0.100
Previous	39.1	40.1	38.8	
Never	53.4	48.8	54.7	
Educational status (%)				
Low	9.2	7.6	9.6	0.001
Medium	17.3	27.3	14.5	
High	69.7	61.1	72.2	
Other	3.8	4.1	3.8	
Physical activity (steps/day)	10,338 (8085, 12,749)	10,696 (8,892, 13,973)	10,127 (7,971, 12,378)	<0.001
Intervention group (%)				
Control	26.2	25.0	26.6	0.931
Self-regulation and motivation	24.4	26.2	23.9	
Stress and emotion regulation	24.6	23.8	24.8	
Combined	24.8	25.0	24.8	
Country				
UK	32.3	14.5	37.3	<0.001
Denmark	37.3	18.0	42.7	
Portugal	30.4	67.4	20.0	
PSS (possible range: 0-16)	6.0 (4.0, 8.0)	5.0 (3.0, 7.0)	6.0 (4.0, 8.0)	0.001
Sleep duration (hours)	7.8 (7.3, 8.4)	7.4 (6.7, 8.1)	7.9 (7.4, 8.4)	<0.001
Sleep onset variability (hours)	1.1 (0.8, 1.5)	1.2 (0.8, 1.7)	1.1 (0.8, 1.5)	0.075

^1^Results presented as median (interquartile range) unless otherwise stated.

^2^BMI, Body Mass Index; PSS, Perceived stress scale.

^3^P-value for sex difference (Wilcoxon rank-sum test or chi-squared test).

At baseline, a higher median HCC was observed among men [55.2 pg/mg (IQR: 26.5, 105.2)] than women [43.2 pg/mg (IQR: 20.3, 106.2); *P=0.032*]. However, higher values were observed for women [53.3 pg/mg (IQR: 24.5, 141.6)] than men [33.3 pg/mg (IQR: 19.3, 97.8); *P=0.008*] at follow-up. Moreover, a higher BW and a lower BF% was observed for men than women at all visits (all *P*<0.001) (**Table 2**), while no consistent differences in BWV was observed (**Table 3**).

**Table 2:** Information on hair cortisol, body weight and body fat percentage among men and women from the NoHoW study.

	All^1^		Men		Women		P^3^
	n	Median (IQR)	n	Median (IQR)	n	Median (IQR)	
**Weight of hair sample (mg)**							
Baseline	786	15.3 (9.4, 21.6)	172	14.7 (8.1, 20.7)	614	15.3 (9.9, 22.0)	0.07
Month 12	614	17.5 (11.3, 22.8)	122	12.1 (8.3, 16.8)	492	19.2 (12.5, 23.5)	<0.001
**HCC^2^ (pg/mg)**							
Baseline	786	45.1 (21.6, 106.2)	172	55.2 (26.5, 105.2)	614	43.2 (20.3, 106.2)	0.032
Month 12	614	50.2 (23.2, 133.8)	122	33.3 (19.3, 97.8)	492	53.3 (24.5, 141.6)	0.008
**Log HCC (pg/mg)**							
Baseline	786	3.8 (3.1, 4.7)	172	4.0 (3.3, 4.7)	614	3.8 (3.0, 4.7)	0.032
Month 12	614	3.9 (3.1, 4.9)	122	3.5 (3.0, 4.6)	492	4.0 (3.2, 5.0)	0.008
**BW (kg)**							
Baseline	786	82.0 (72.7, 94.7)	172	89.1 (79.2, 101.8)	614	80.1 (70.4, 91.6)	<0.001
Month 6	773	81.9 (71.8, 93.4)	168	88.6 (78.3, 102.4)	605	79.5 (69.7, 91.0)	<0.001
Month 12	713	81.7 (72.4, 93.9)	158	88.3 (78.8, 102.2)	555	80.0 (70.0, 92.1)	<0.001
Month 18	668	82.1 (73.2, 94.9)	145	88.8 (79.2, 102.3)	523	80.2 (71.2, 93.6)	<0.001
**BF%**							
Baseline	774	39.0 (33.2, 45.0)	167	32.1 (27.1, 37.5)	607	41.1 (35.6, 46.5)	<0.001
Month 6	765	38.7 (32.9, 44.5)	167	32.6 (25.6, 37.5)	598	40.0 (35.2, 46.0)	<0.001
Month 12	701	38.7 (32.8, 44.4)	157	30.8 (25.8, 36.1)	544	40.8 (35.3, 45.8)	<0.001
Month 18	657	39.1 (33.4, 45.4)	145	32.4 (27.2, 36.6)	512	41.4 (36.3, 46.4)	<0.001

^1^Results presented as median (interquartile range) unless otherwise stated.

^2^BF%, Body fat percentage;BW, body weight; HCC, hair cortisol concentration; AQR, interquartile range.

^3^P-value for sex difference (Wilcoxon rank-sum test or chi-squared test).

**Table 3:** Number of weight measurements from Fitbit Aria scales and body weight variability among men and women from the NoHoW study.

	All^1^		Men		Women		P ^3^
	n	Median (IQR)	n	Median (IQR)	n	Median (IQR)	
**Number BW^2^ measurements**							
Baseline to month 6	755	79.0 (51.0, 123.0)	163	94.0 (51.0, 136.0)	592	76.0 (50.0, 121.5)	0.031
Baseline to month 12	734	142.5 (85.0, 227.0)	159	158.0 (86.0, 229.0)	575	140.0 (85.0, 224.0)	0.237
Baseline to month 18	677	198.0 (121.0, 317.0)	146	211.5 (120.0, 309.0)	531	186.0 (121.0, 323.0)	0.436
Month 12 to month 18	593	58.0 (33.0, 108.0)	126	56.0 (33.0, 100.0)	467	59.0 (33.0, 108.0)	0.741
**RMSE (%)**							
Baseline to month 6	755	1.1 (0.9, 1.4)	163	1.0 (0.9, 1.3)	592	1.1 (0.9, 1.4)	0.14
Baseline to month 12	734	1.4 (1.1, 1.8)	159	1.4 (1.1, 1.8)	575	1.4 (1.1, 1.8)	0.616
Baseline to month 18	677	1.6 (1.3, 2.2)	146	1.5 (1.2, 2.1)	531	1.6 (1.3, 2.2)	0.074
Month 12 to month 18	593	1.0 (0.8, 1.3)	126	1.0 (0.8, 1.3)	467	1.1 (0.8, 1.3)	0.163
**NLMD (%)**							
Baseline to month 6	755	0.7 (0.6, 0.8)	163	0.6 (0.6, 0.8)	592	0.7 (0.6, 0.8)	0.018
Baseline to month 12	734	0.8 (0.6, 0.9)	159	0.7 (0.6, 0.9)	575	0.8 (0.7, 0.9)	0.031
Baseline to month 18	677	0.8 (0.7, 1.0)	146	0.8 (0.7, 1.0)	531	0.8 (0.7, 1.0)	0.204
Month 12 to month 18	593	0.6 (0.5, 0.8)	126	0.6 (0.5, 0.8)	467	0.6 (0.5, 0.8)	0.385

^1^Results presented as median (interquartile range) unless otherwise stated.

^2^BW, Body weight; IQR, interquartile range; NLMD; non-linear mean deviation; RMSE, root-mean-square deviation.

^3^P-value for sex difference (Wilcoxon rank-sum test or chi-squared test).

We found no associations between baseline log HCC and subsequent change in BW or BF% during 6, 12 or 18 months. Moreover, while crude models showed baseline log HCC to be associated with higher subsequent BWV during the first 6 and 12 months, using both RMSE and NLMD, no statistically significant associations were observed after adjustment for covariates (**Table 4**). Likewise, we found no associations between 12-month concurrent changes in log HCC and BW, BF% or BWV (**Table 5**). Finally, while no association between initial 12-month changes in log HCC and subsequent change in BW and BF% was found, an initial increase in log HCC was associated with a higher subsequent BWV. However, after adjusting for potential confounding factors this association was only statistically significant using the NLMD method (β: 0.02% per unit increase in log HCC [95% CI: 0.00, 0.04]; *P*=0.016) (**Table 6**).

**Table 4:** Association between baseline log hair cortisol concentration (pg/mg) and subsequent 6-, 12-, and 18-month weight loss maintenance, change in body fat percentage and body weight variability.

	Month 6				Month 12				Month 18			
	n	β^1^	(95% CI)	P	n	β	(95% CI)	P	n	β	(95% CI)	P
**ΔBW^2^ (kg)**												
Crude^3^	773	-0.22	(-0.50, 0.07)	0.134	713	-0.06	(-0.45, 0.33)	0.766	668	0.02	(-0.42, 0.47)	0.913
Adjusted^4^	773	-0.15	(-0.44, 0.14)	0.297	713	-0.00	(-0.40, 0.39)	0.984	668	0.03	(-0.42, 0.47)	0.907
**ΔBF%**												
Crude	755	-0.24	(-.055, 0.07)	0.128	691	-0.03	(-0.36, 0.30)	0.852	646	-0.06	(-0.39, 0.27)	0.737
Adjusted	755	-0.03	(-0.33, 0.28)	0.863	691	0.18	(-0.14, 0.50)	0.271	646	0.15	(-0.16, 0.47)	0.342
**RMSE (%)**												
Crude	755	0.04	(0.01, 0.08)	0.023	734	0.05	(0.01, 0.10)	0.025	677	0.05	(-0.01, 0.11)	0.111
Adjusted	755	0.03	(-0.01, 0.07)	0.101	734	0.04	(-0.01, 0.08)	0.109	677	0.04	(-0.02, 0.09)	0.250
**NLMD (%)**												
Crude	755	0.02	(0.00, 0.04)	0.029	734	0.02	(0.00, 0.04)	0.036	677	0.02	(-0.00, 0.04)	0.082
Adjusted	755	0.01	(-0.00, 0.03)	0.119	734	0.01	(-0.00, 0.03)	0.110	677	0.01	(-0.00, 0.03)	0.113

^1^Results presented 6, 12 and 18-month change in outcomes (95% CI) per additional unit higher baseline log hair cortisol.

^2^BF%, Body fat percentage; BW, body weight; NLMD, non-linear mean deviation; RMSE, root-mean-square deviation.

^3^Model with exposure, outcome and baseline measure of outcome, only.

^4^Adjusted for physical activity (steps/day), smoking status, educational level, sex, alcohol, age, height, intervention status, country and initial weight loss.

**Table 5:** Association between 12-month concurrent changes in log HCC and body weight, body fat percentage and 12-month body weight variability.

	n	β^1^	(95% CI)	P
**ΔBW^2^ (kg)**				
Crude^3^	612	0.08	(-0.36, 0.53)	0.719
Adjusted^4^	612	0.01	(-0.44, 0.46)	0.968
**ΔBF%**				
Crude	597	0.04	(-0.33, 0.41)	0.846
Adjusted	597	-0.05	(-0.41, 0.30)	0.781
**RMSE (%)**				
Crude	588	-0.00	(-0.05, 0.05)	0.940
Adjusted	588	-0.03	(-0.08, 0.03)	0.320
**NLMD (%)**				
Crude	588	0.02	(-0.00, 0.03)	0.110
Adjusted	588	0.01	(-0.01, 0.03)	0.428

^1^Results presented as change in outcomes (95% CI) between baseline and month 12 per additional unit of 12-month concurrent change in log hair cortisol.

^2^BF%, Body fat percentage; BW, body weight; NLMD, non-linear mean deviation; RMSE, root-mean-square deviation.

^3^Model with exposure, outcome and baseline measures of both, only.

^4^Adjusted for physical activity (steps/day), smoking status, educational level, sex, alcohol, age, height, intervention status, country and initial weight loss.

**Table 6:** Association between initial 12-month changes in log HCC and subsequent change in body weight, body fat percentage and variability in body weight between month 12 and month 18.

	n	β^1^	(95% CI)	P
**ΔBW^2^ (kg)**				
Crude^3^	568	0.08	(-0.21, 0.37)	0.593
Adjusted^4^	568	-0.04	(-0.33, 0.24)	0.760
**ΔBF%**				
Crude	556	0.33	(0.05, 0.61)	0.021
Adjusted	556	0.24	(-0.05, 0.52)	0.100
**RMSE (%)**				
Crude	494	0.34	(-0.00, 0.07)	0.057
Adjusted	494	0.03	(-0.01, 0.06)	0.149
**NLMD (%)**				
Crude	494	0.02	(0.01, 0.04)	0.008
Adjusted	494	0.02	(0.00, 0.04)	0.016

^1^Results presented as change in outcomes (95% CI) between month moth 12 and 18 per additional unit change in log hair cortisol between baseline and month 12.

^2^BF%, Body fat percentage; BW, body weight; NLMD, non-linear mean deviation; RMSE, root-mean-square deviation.

^3^Model with exposure, outcome and baseline measures of both, only.

^4^Adjusted for physical activity (steps/day), smoking status, educational level, sex, alcohol, age, height, intervention status, country and initial weight loss.

None of the analyses showed evidence of interaction between log HCC (baseline level or change) and sex or intervention status (all p-values > 0.05).

### Sensitivity Analyses

Additional adjustment for the PSS, sleep duration and/or sleep onset variability gave essentially similar associations (**Supplementary Tables 1**–**3**), as did adjustment for weight of the hair sample, use of hair colouring products and hair washing frequency (**Supplementary Tables 4**–**6**), indicating no or limited confounding from these factors. Analyses excluding participants with less than 10 mg of hair also gave similar associations (**Supplementary Tables 7**–**9**). Likewise, analyses excluding participants treated with any form of medication gave almost identical results (**Supplementary Tables 10**–**12**).

## Discussion

In a large WLM trial of European men and women, we found no evidence of association between baseline HCC and subsequent change in BW, change in BF% or BWV when controlling for lifestyle and demographic factors. Likewise, no associations between 12-month concurrent development in HCC and outcome measures were observed, but an initial 12-month increase in HCC was associated with a higher subsequent BWV between month 12 and 18.

While a large body of evidence has confirmed a cross-sectional association between HCC and adiposity (21–24), to our knowledge, no previous studies have explored the relationship between HCC and WLM. However, similar studies have been conducted based on self-reported information on psychosocial stress. For instance, Brantley and colleagues (2014) found perceived stress to be associated with weight regain in a WLM trial of 1,025 men and women (10), and some intervention studies have suggested that stress management can facilitate WL or weight maintenance (41–43). However, our results suggest that an increased long-term cortisol secretion was not the primary driver of these associations.

Likewise, we found no previous studies examining the association between HCC and BWV, but results from 4,774 participants from the Look AHEAD study suggested an association between mental health or depressive symptoms and BWV (44). This may explain the association between initial 12 month increase in HCC and subsequent 6 month BWV observed in the present study as van Manen and colleagues (2019) have shown HCC to be directly associated to subjective measures of psychological distress (45). Another explanation for the association between an increased HCC and a subsequent higher BWV observed in our study may be a tendency towards more irregular eating patterns when stress levels increase (13, 14). Such an eating pattern could theoretically lead to a higher degree of fluctuation in calorie intake, without necessarily affecting the average intake. However, it is worth mentioning, that although some studies have shown a high degree of BWV to be associated with both future weight gain, morbidity and mortality (16, 46), these studies are often based on too few BW measurements to get an accurate estimate of BWV and thus any clinical significance of our results is uncertain (30).

The present study has several strengths, including the use of data from a large WLM trial with repeated information on HCC and outcome measures, and the use of prospective analyses which reduces the risk of reverse causality. Moreover, in addition to data collected at the four centre visits, all participants were instructed to weigh themselves on the validated Fitbit Aria scale (27) at least twice per week for the 18 month duration of the study, providing us with detailed measures of BWV. We also had verified information on several lifestyle factors, including objectively measured physical activity and sleep habits, allowing us to adjust for potential influence from these factors.

The study also has some limitations. For the analyses of HCC, we aimed to analyse 10-30 mg of hair. However, especially among the male participants, the collected samples did not consistently contain sufficient hair to meet this criterion. As we found a direct correlation between the weight of the hair samples and HCC, this may have affected the measured values. However, as we found almost identical associations estimates in sensitivity analyses adjusted for weight of the hair sample, and when excluding individuals with less than 10 mg of hair available, it seems unlikely that this had a substantial influence on the observed associations. The detailed BW information, collected twice weekly using the validated Fitbit Aria scale, gave us a detailed measure of BWV during the trial, but we do not know exactly what tissues or circumstances were actually fluctuating (e.g., fat mass, water, glycogen, weight of gut content, clothing and/or time of day), and indeed it is likely that the majority of fluctuating body weight relates to acute fluctuation in total body water (47). In addition, although we adjusted our analyses for several potential confounding factors, we cannot exclude that some unmeasured or residual confounding has remained. As an example, although we did perform sensitivity analyses excluding participants currently treated with any form of medication, which did not change the observed associations, we did not specifically ask participants on their use of corticosteroids. Moreover, we did not adjust for multiple testing, and thus results for the secondary outcomes should be interpreted with caution due to the risk of false positive associations.

We conducted analyses of the association between HCC and outcome measures using three different analytical approaches, as each of them have their own set of limitations. In analyses of baseline HCC and subsequent change in adiposity or BWV, the temporal separation between exposure and outcome measures is a clear strength. However, when using this approach, a comparison of different individuals who vary in HCC is made. As HCC has a high genetic component (48, 49) and people are likely to vary according to other factors than their HCC, confounding may be a more pronounced problem when using this strategy. Analysing concurrent 12-month changes in two measures is subject to the same limitations as a cross-sectional design, and thus it is not possible to determine causality. In contrast, analysing the association between preceding changes in HCC during the first 12-month period and outcomes during the subsequent 6-month period is a strength in terms of keeping a prospective structure in the analyses (e.g. temporally separating the change in HCC from the change in outcome measures). However, this approach also introduces a limitation, as the potential effect of changes in HCC on adiposity may have occurred in the same 12-month period or shortly thereafter.

Finally, our results primarily originate from individuals with overweight or obesity. All subjects had achieved a clinically significant WL prior to enrolment, and ¾ of the participants received a digital intervention based on self-regulation, motivation, and/or emotion regulation tools designed to improve WLM. Thus, generalisation to the general population should be done with caution.

In conclusion, our data suggest that an association between HCC and WLM is either absent or marginal. An initial increase in HCC may predict a slightly higher subsequent BWV, but the clinical importance of this is unknown. Additional longitudinal studies are needed to establish the causal direction and underlying mechanisms of the association between HCC and adiposity.

## Data Availability Statement

The datasets presented in this study can be found in online repositories. The names of the repository/repositories and accession number(s) can be found below: https://easo.org/the-nohow-dataset/.

## Ethics Statement

The studies involving human participants were reviewed and approved by local institutional ethics committees at the Universities of Leeds (17-0082; 27 February 2017), Lisbon (17/2016; 20 February 2017) and the Capital Region of Denmark (H-16030495; 8 March 2017). The patients/participants provided their written informed consent to participate in this study.

## Author Contributions

Conceptualization: SL and BH. Data collection: SL, M-LM, AP, SS, CD, IS, JE, RO’D, JT, and JM. Analysis of biological samples: GC, CL, and NJ. Data curation: SL, GH, and JT. Formal statistical analysis: SL. Writing: SL, JT, GC, CL, NJ, M-LM, GH, RO’D, JM, CD, SS, IS, JE, AP, RS, and BH. All authors contributed to the article and approved the submitted version.

## Funding 

The NoHoW study has received funding from the European Union’s Horizon 2020 Research and Innovation Programme (grant agreement number 643309). The present ancillary study was supported by the Danish Health Foundation (Helsefonden) (grant agreement number 20-B-0405). The Parker Institute is supported by a core grant from the Oak Foundation (grant agreement number OCAY-18-774-OFIL).

## Conflict of Interest

RS consults for Slimming World UK *via* Consulting Leeds, a wholly owned subsidiary of the University of Leeds.

The remaining authors declare that the research was conducted in the absence of any commercial or financial relationships that could be construed as a potential conflict of interest.

## Publisher’s Note

All claims expressed in this article are solely those of the authors and do not necessarily represent those of their affiliated organizations, or those of the publisher, the editors and the reviewers. Any product that may be evaluated in this article, or claim that may be made by its manufacturer, is not guaranteed or endorsed by the publisher.
