# Supplementary material for: Hair Cortisol Concentration, Weight Loss Maintenance and Body Weight Variability: A Prospective Study Based on Data From the European NoHoW Trial
Source: Front Endocrinol (Lausanne). 2021 Sep 29;12:655197. doi: 10.3389/fendo.2021.655197 (PMC8511813; doi:10.3389/fendo.2021.655197)
Supplement: Supplementary file 1 [file DataSheet_1.docx]

**Supplementary Text 1.** Hair cortisol concentration and long-term weight loss maintenance: a NoHoW analysis plan. Date: 12/06/2020

Sofus C. Larsen, Jake Turicchi, Glostrup representatives, Marie-Louise K. Mikkelsen, Graham Horgan, Ruairi O'Driscoll, Joanna Michalowska, Cristiana Duarte, Sarah Scott, Inês Santos, Jorge Encantado, Antonio L Palmeira, R. James Stubbs, Berit L. Heitmann

**Background**

Measurement of cortisol in blood, saliva or urine are often used as biological markers of stress. An important limitation of these measures is that they provide information of current cortisol level, and consequently are an expression of acute, rather than chronic, stress. In contrast to these measures, hair cortisol concentration (HCC) is biomarker for assessing chronic stress, by reflecting cortisol exposure during the period of hair growth.

Cortisol plays a role in many hormonal and metabolic reactions that may lead to weight regain or prevent further weight loss (Tomiyama, 2019). Several studies have shown that HCC is related to a higher BMI at a cross-sectional level (Larsen et al., 2016; Stalder et al., 2017; Stalder et al., 2012), but the role of chronic stress in long-term weight loss maintenance is largely unknown.

The aim of this study is to examine the longitudinal associations between HCC and weight loss maintenance, change in body composition and body weight variability among the NoHoW participants.

**Hypotheses**

Main hypothesis:

- Baseline HCC and change in HCC is directly associated with weight regain

Secondary hypotheses:

- Baseline HCC and change in HCC is directly associated with change body fat percentage
- Baseline HCC and change in HCC is directly associated with body weight variability

**Material and methods**

The present study is an ancillary study based on data collected on participants from NoHoW trial (Scott et al., 2019). Analyses will include all NoHoW-participants with information on predefined variables.

**Measures**

We will include baseline and follow-up information on HCC and outcome variables (body weight, body fat percentage, body weight variability). Change in body weight and body composition will be accessed using measured information from study visits, while body weight variability will be accessed using home monitored information from the Fitbit Aria scales. Finally, we will include the following preselected covariates: initial weight loss, smoking status, frequency of alcohol consumption, physical activity, education, age, intervention status (arm allocation), centre (country) sex, perceived stress, hair washing frequency, use of hair dye, weight of hair sample, objectively measured sleep duration and sleep onset variability.

**Statistical analyses**

Multivariate linear regression analyses will be conducted to assess the associations between baseline HCC and subsequent 6, 12 and 18-month change in body weight and composition, in addition to body weight variability over the same time periods. We will also explore the association between concurrent 12-month changes in HCC and outcome measures. Finally, the association between 12-month change in HCC and subsequent change in outcome measures from month 12 to month 18 will be explored.

First, crude models, including information on outcome, exposure and baseline measure of outcome, only, will be conducted. Secondly, adjusted analyses with added information on initial weight loss, smoking status, frequency of alcohol consumption, physical activity, education, age, intervention status (arm allocation) and sex as potential confounding factors will be carried out.

Sex and intervention interactions will be tested in all analyses by adding product terms to the models and subgroup analyses will be conducted if appropriate.

All statistical tests will be two-tailed with a significance level at 0.05. Analyses will be performed using Stata SE 14 (StataCorp LP, College Station, Texas, USA).

*Sensitivity analyses*

To get associations independent of perceived stress, sensitivity analyses additionally adjusted for the perceived stress scale will be conducted. Moreover, since stress is closely related to sleep habits (Yang et al., 2018), and we have previously shown sleep onset variability is associated with weight regain and increased body fat percentage among the NoHoW participants (Larsen et al., 2020), sensitivity analyses will be conducted adjusting for sleep duration and sleep onset variability. Finally, sensitivity analyses adjusted for hair washing frequency, use of hair dye and weight of the hair sample will be conducted.

**References**

Larsen SC, Fahrenkrug J, Olsen NJ et al. Association between Hair Cortisol Concentration and Adiposity Measures among Children and Parents from the "Healthy Start" Study. PLoS One. 2016;11(9):e0163639.

Larsen SC, Horgan G, Mikkelsen MLK et al. Consistent sleep onset and maintenance of body weight after weight loss: An analysis of data from the NoHoW trial. PLOS Medicine 2020; 17, e1003168.

Scott SE, Duarte C, Encantado J, et al. The NoHoW protocol: a multicentre 2×2 factorial randomised controlled trial investigating an evidence-based digital toolkit for weight loss maintenance in European adults. BMJ Open. 2019;9(9):e029425.

Stalder T, Steudte S, Alexander N, et al. Cortisol in hair, body mass index and stress-related measures. Biol Psychol. 2012;90(3):218-223.

Stalder T, Steudte-Schmiedgen S, Alexander N, et al. Stress-related and basic determinants of hair cortisol in humans: A meta-analysis. Psychoneuroendocrinology. 2017;77:261-274.

Tomiyama AJ. Stress and Obesity. Annu Rev Psychol. 2019;70(1):703-718.

Yang B, Wang Y, Cui F, et al. Association between insomnia and job stress: a meta-analysis. Sleep Breath. 2018;22(4):1221-1231.


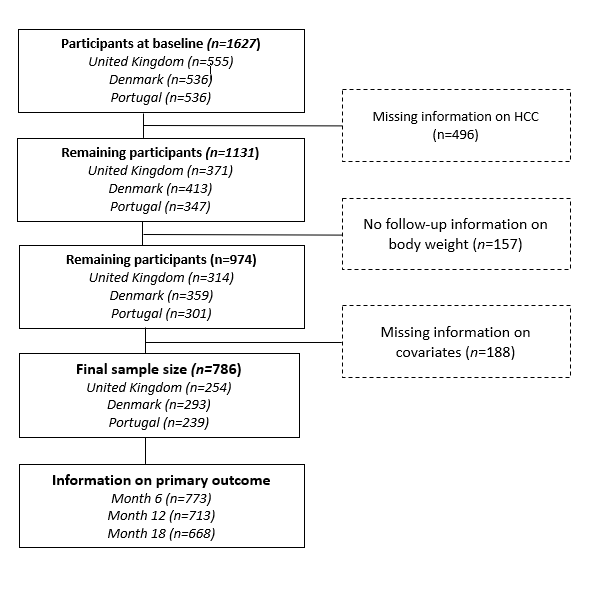


**Supplementary Figure 1.** Flowchart illustrating the inclusion/exclusion of individuals in the NoHoW study.

**Supplementary Table 1.** Association between baseline log hair cortisol concentration (pg/mg) and subsequent 6-, 12-, and 18-month weight loss maintenance, change in body fat percentage and body weight variability (adjusted model with added information on PSS, sleep duration and/or sleep onset variability)

|  | **Month 6** | | | | **Month 12** | | | | **Month 18** | | | |
| --- | --- | --- | --- | --- | --- | --- | --- | --- | --- | --- | --- | --- |
|  | **n** | **β^1^** | **(95% CI)** | **P** | **n** | **β** | **(95% CI)** | **P** | **n** | **β** | **(95% CI)** | **P** |
| **ΔBW^2^ (kg)** |  |  |  |  |  |  |  |  |  |  |  |  |
| + PSS | 748 | -0.14 | (-0.43, 0.16) | 0.366 | 691 | -0.01 | (-0.42, 0.39) | 0.944 | 649 | 0.04 | (-0.41, 0.49) | 0.853 |
| + sleep duration | 773 | -0.16 | (-0.45, 0.13) | 0.282 | 713 | -0.00 | (-0.40, 0.39) | 0.989 | 668 | 0.03 | (-0.41, 0.47) | 0.901 |
| + sleep onset variability | 773 | -0.16 | (-0.45, 0.13) | 0.280 | 713 | -0.02 | (-0.41, 0.37) | 0.918 | 668 | 0.01 | (-0.44, 0.46) | 0.978 |
| + all the above | 748 | -0.14 | (-0.44, 0.15) | 0.345 | 691 | -0.03 | (-0.43, 0.37) | 0.893 | 649 | 0.04 | (-0.41, 0.49) | 0.868 |
| **ΔBF%** |  |  |  |  |  |  |  |  |  |  |  |  |
| + PSS | 730 | -0.01 | (-0.32, 0.30) | 0.951 | 670 | 0.18 | (-0.15, 0.50) | 0.295 | 627 | 0.18 | (-0.15, 0.50) | 0.281 |
| + sleep duration | 755 | -0.03 | (-0.34, 0.27) | 0.835 | 691 | 0.18 | (-0.14, 0.50) | 0.274 | 646 | 0.15 | (-0.17, 0.47) | 0.346 |
| + sleep onset variability | 755 | -0.04 | (-0.34, 0.27) | 0.821 | 691 | 0.17 | (-0.15, 0.50) | 0.290 | 646 | 0.14 | (-0.17, 0.46) | 0.370 |
| + all the above | 730 | -0.02 | (-0.33, 0.29) | 0.903 | 670 | 0.17 | (-0.16, 0.50) | 0.309 | 627 | 0.17 | (-0.16, 0.49) | 0.307 |
| **RMSE (%)** |  |  |  |  |  |  |  |  |  |  |  |  |
| + PSS | 732 | 0.03 | (-0.01, 0.06) | 0.157 | 712 | 0.03 | (-0.01, 0.08) | 0.188 | 656 | 0.03 | (-0.03, 0.09) | 0.351 |
| + sleep duration | 755 | 0.03 | (-0.01, 0.07) | 0.105 | 734 | 0.04 | (-0.01, 0.08) | 0.115 | 677 | 0.03 | (-0.03, 0.10) | 0.262 |
| + sleep onset variability | 755 | 0.03 | (-0.01, 0.07) | 0.106 | 734 | 0.04 | (-0.01, 0.08) | 0.109 | 677 | 0.04 | (-0.02, 0.10) | 0.244 |
| + all the above | 732 | 0.02 | (-0.01, 0.06) | 0.181 | 712 | 0.03 | (-0.02, 0.08) | 0.199 | 656 | 0.03 | (-0.03, 0.09) | 0.365 |
| **NLMD (%)** |  |  |  |  |  |  |  |  |  |  |  |  |
| + PSS | 732 | 0.01 | (-0.00, 0.03) | 0.160 | 712 | 0.01 | (-0.01, 0.03) | 0.174 | 656 | 0.01 | (-0.01, 0.03) | 0.155 |
| + sleep duration | 755 | 0.01 | (-0.00, 0.03) | 0.120 | 734 | 0.01 | (-0.00, 0.03) | 0.118 | 677 | 0.01 | (-0.01, 0.03) | 0.161 |
| + sleep onset variability | 755 | 0.01 | (-0.00. 0.03) | 0.120 | 734 | 0.01 | (-0.00, 0.03) | 0.114 | 677 | 0.01 | (-0.01, 0.03) | 0.156 |
| + all the above | 732 | 0.01 | (-0.01, 0.03) | 0.176 | 712 | 0.01 | (-0.01, 0.03) | 0.194 | 656 | 0.01 | (-0.01, 0.03) | 0.229 |

^1^ Results presented 6, 12 and 18-month change in outcomes (95% CI) per additional unit higher baseline log hair cortisol

^2^ Abbreviations: Body fat percentage (BF%), body weight (BW), non-linear mean deviation (NLMD), Perceived stress (PSS), root-mean-square deviation (RMSE).

^3^ Model with exposure, outcome and baseline measure of outcome, only.

^4^ Adjusted for physical activity (steps/day), smoking status, educational level, sex, alcohol, age, height, intervention status, country, initial weight loss (added information on PSS, sleep duration sleep onset variability or all factors combined).

**Supplementary Table 2.** Association between 12-month concurrent changes in log HCC and body weight, body fat percentage and 12-month body weight variability (adjusted model with added information on PSS, sleep duration and/or sleep onset variability)

|  | **n** | **β^1^** | **(95% CI)** | **P** |
| --- | --- | --- | --- | --- |
| **ΔBW^2^ (kg)** |  |  |  |  |
| + PSS | 592 | 0.03 | (-0.43, 0.49) | 0.895 |
| + sleep duration | 612 | -0.03 | (-0.48, 0.42) | 0.894 |
| + sleep onset variability | 612 | 0.03 | (-0.42, 0.48) | 0.887 |
| + all the above | 592 | 0.01 | (-0.45, 0.47) | 0.959 |
| **ΔBF%** |  |  |  |  |
| + PSS | 577 | -0.03 | (-0.39, 0.34) | 0.887 |
| + sleep duration | 597 | -0.06 | (-0.42, 0.30) | 0.741 |
| + sleep onset variability | 597 | -0.04 | (-0.40, 0.31) | 0.817 |
| + all the above | 577 | -0.03 | (-0.39, 0.34) | 0.890 |
| **RMSE (%)** |  |  |  |  |
| + PSS | 570 | -0.03 | (-0.08, 0.02) | 0.214 |
| + sleep duration | 588 | -0.02 | (-0.08, 0.03) | 0.346 |
| + sleep onset variability | 588 | -0.03 | (-0.08, 0.03) | 0.327 |
| + all the above | 570 | -0.03 | (-0.08, 0.02) | 0.243 |
| **NLMD (%)** |  |  |  |  |
| + PSS | 570 | 0.01 | (-0.01, 0.02) | 0.551 |
| + sleep duration | 588 | 0.01 | (-0.01, 0.03) | 0.407 |
| + sleep onset variability | 588 | 0.01 | (-0.01, 0.03) | 0.410 |
| + all the above | 570 | 0.01 | (-0.01, 0.03) | 0.488 |

^1^ Results presented as change in outcomes (95% CI) between baseline and month 12 per additional unit of 12-month concurrent change in log hair cortisol

^2^ Abbreviations: Body fat percentage (BF%), body weight (BW), non-linear mean deviation (NLMD), root-mean-square deviation (RMSE).

^3^ Model with exposure, outcome and baseline measures of both, only.

^4^ Adjusted for physical activity (steps/day), smoking status, educational level, sex, alcohol, age, height, intervention status, country and initial weight loss

**Supplementary Table 3.** Association between initial 12-month changes in log HCC and subsequent change in body weight, body fat percentage and variability in body weight between month 12 and month 18 (adjusted model with added information on PSS, sleep duration and/or sleep onset variability)

|  | **n** | **β^1^** | **(95% CI)** | **P** |
| --- | --- | --- | --- | --- |
| **ΔBW^2^ (kg)** |  |  |  |  |
| + PSS | 550 | -0.05 | (-0.34, 0.25) | 0.761 |
| + sleep duration | 568 | -0.05 | (-0.34, 0.23) | 0.717 |
| + sleep onset variability | 568 | -0.04 | (-0.32, 0.25) | 0.790 |
| + all the above | 550 | -0.05 | (-0.34, 0.25) | 0.748 |
| **ΔBF%** |  |  |  |  |
| + PSS | 538 | 0.25 | (-0.04, 0.54) | 0.090 |
| + sleep duration | 556 | 0.24 | (-0.04, 0.52) | 0.095 |
| + sleep onset variability | 556 | 0.24 | (-0.04, 0.53) | 0.088 |
| + all the above | 538 | 0.27 | (-0.02, 0.56) | 0.071 |
| **RMSE (%)** | |  |  |  |
| + PSS | 480 | 0.02 | (-0.01, 0.06) | 0.176 |
| + sleep duration | 494 | 0.03 | (-0.01, 0.06) | 0.139 |
| + sleep onset variability | 494 | 0.03 | (-0.01, 0.06) | 0.159 |
| + all the above | 480 | 0.02 | (-0.01, 0.06) | 0.171 |
| **NLMD (%)** | |  |  |  |
| + PSS | 480 | 0.02 | (0.00, 0.04) | 0.014 |
| + sleep duration | 494 | 0.02 | (0.00, 0.04) | 0.014 |
| + sleep onset variability | 494 | 0.02 | (0.00, 0.04) | 0.016 |
| + all the above | 494 | 0.02 | (0.01, 0.04) | 0.012 |

^1^ Results presented as change in outcomes (95% CI) between month moth 12 and 18 per additional unit change in log hair cortisol between baseline and month 12.

^1^ Abbreviations: Body fat percentage (BF%), body weight (BW), non-linear mean deviation (NLMD), root-mean-square deviation (RMSE).

^3^ Model with exposure and outcome and baseline measures of both, only.

^4^ Adjusted for physical activity (steps/day), smoking status, educational level, sex, alcohol, age, height, intervention status, country and initial weight loss

**Supplementary Table 4.** Association between baseline log hair cortisol concentration (pg/mg) and subsequent 6-, 12-, and 18-month weight loss maintenance, change in body fat percentage and body weight variability (adjusted model with added information weight of hair sample, use of hair colouring products and hair washing frequency)

|  | **Month 6** | | | | **Month 12** | | | | **Month 18** | | | |
| --- | --- | --- | --- | --- | --- | --- | --- | --- | --- | --- | --- | --- |
|  | **n** | **β^1^** | **(95% CI)** | **P** | **n** | **β** | **(95% CI)** | **P** | **n** | **β** | **(95% CI)** | **P** |
| **ΔBW^2^ (kg)** |  |  |  |  |  |  |  |  |  |  |  |  |
| Adjusted | 768 | -0.18 | (-0.47, 0.12) | 0.239 | 709 | -0.03 | (-0.44, 0.38) | 0.884 | 663 | -0.02 | (-0.48, 0.43) | 0.917 |
| **ΔBF%** |  |  |  |  |  |  |  |  |  |  |  |  |
| Adjusted | 751 | -0.04 | (-0.35, 0.27) | 0.817 | 688 | 0.13 | (-0.20, 0.46) | 0.449 | 642 | 0.12 | (-0.21, 0.44) | 0.483 |
| **RMSE (%)** |  |  |  |  |  |  |  |  |  |  |  |  |
| Adjusted | 751 | 0.03 | (-0.01, 0.07) | 0.106 | 730 | 0.03 | (-0.02, 0.08) | 0.213 | 673 | 0.03 | (-0.03, 0.09) | 0.376 |
| **NLMD (%)** |  |  |  |  |  |  |  |  |  |  |  |  |
| Adjusted | 751 | 0.02 | (-0.00, 0.03) | 0.082 | 730 | 0.01 | (-0.00, 0.03) | 0.135 | 673 | 0.01 | (-0.01, 0.03) | 0.186 |

^1^ Results presented 6, 12 and 18-month change in outcomes (95% CI) per additional unit higher baseline log hair cortisol

^2^ Abbreviations: Body fat percentage (BF%), body weight (BW), non-linear mean deviation (NLMD), root-mean-square deviation (RMSE).

^3^ Model with exposure, outcome and baseline measure of outcome, only.

^4^ Adjusted for physical activity (steps/day), smoking status, educational level, sex, alcohol, age, height, intervention status, initial weight loss, country, weight of hair sample, use of hair colouring products and hair washing frequency.

**Supplementary Table 5.** Association between 12-month concurrent changes in log HCC and body weight, body fat percentage and 12-month body weight variability (adjusted model with added information on weight of hair samples, use of hair colouring products and hair washing frequency)

|  | **n** | **β^1^** | **(95% CI)** | **P** |
| --- | --- | --- | --- | --- |
| **ΔBW^2^ (kg)** |  |  |  |  |
| Adjusted | 608 | -0.10 | -0.58, 0.39 | 0.698 |
| **ΔBF%** |  |  |  |  |
| Adjusted | 593 | -0.09 | -0.47, 0.29 | 0.641 |
| **RMSE (%)** |  |  |  |  |
| Adjusted | 582 | -0.02 | -0.07, 0.04 | 0.538 |
| **NLMD (%)** |  |  |  |  |
| Adjusted | 582 | 0.01 | -0.01, 0.03 | 0.378 |

^1^ Results presented as change in outcomes (95% CI) between baseline and month 12 per additional unit of 12-month concurrent change in log hair cortisol

^2^ Abbreviations: Body fat percentage (BF%), body weight (BW), non-linear mean deviation (NLMD), root-mean-square deviation (RMSE).

^3^ Model with exposure, outcome and baseline measures of both, only.

^4^ Adjusted for physical activity (steps/day), smoking status, educational level, sex, alcohol, age, height, intervention status, initial weight loss, country, weight of hair samples, use of hair colouring products and hair washing frequency.

**Supplementary Table 6.** Association between initial 12-month changes in log HCC and subsequent change in body weight, body fat percentage and variability in body weight between month 12 and month 18 (adjusted model with added information on weight of hair samples, use of hair colouring products and hair washing frequency)

|  | **n** | **β^1^** | **(95% CI)** | **P** |
| --- | --- | --- | --- | --- |
| **ΔBW^2^ (kg)** |  |  |  |  |
| Adjusted | 564 | 0.07 | -0.24, 0.38 | 0.643 |
| **ΔBF%** |  |  |  |  |
| Adjusted | 552 | 0.23 | -0.07, 0.53 | 0.138 |
| **RMSE (%)** |  |  |  |  |
| Adjusted | 490 | 0.03 | -0.01, 0.07 | 0.148 |
| **NLMD (%)** |  |  |  |  |
| Adjusted | 490 | 0.02 | 0.00, 0.04 | 0.021 |

^1^ Results presented as change in outcomes (95% CI) between month moth 12 and 18 per additional unit change in log hair cortisol between baseline and month 12.

^1^ Abbreviations: Body fat percentage (BF%), body weight (BW), non-linear mean deviation (NLMD), root-mean-square deviation (RMSE).

^3^ Model with exposure and outcome and baseline measures of both, only.

^4^ Adjusted for physical activity (steps/day), smoking status, educational level, sex, alcohol, age, height, intervention status, initial weight loss, country, weight of hair samples, use of hair colouring products and hair washing frequency.

**Supplementary Table 7.** Association between baseline log hair cortisol concentration (pg/mg) and subsequent 6-, 12-, and 18-month weight loss maintenance, change in body fat percentage and body weight variability (participants with less than 10 pg/mg hair excluded)

|  | **Month 6** | | | | **Month 12** | | | | **Month 18** | | | |
| --- | --- | --- | --- | --- | --- | --- | --- | --- | --- | --- | --- | --- |
|  | **n** | **β^1^** | **(95% CI)** | **P** | **n** | **β** | **(95% CI)** | **P** | **n** | **β** | **(95% CI)** | **P** |
| **ΔBW^2^ (kg)** |  |  |  |  |  |  |  |  |  |  |  |  |
| Crude^3^ | 565 | -0.22 | (-0.55, 0.11) | 0.192 | 520 | -0.07 | (-0.53, 0.40) | 0.781 | 483 | 0.09 | (-0.42, 0.61) | 0.722 |
| Adjusted^4^ | 565 | -0.18 | (-0.52, 0.16) | 0.294 | 520 | -0.06 | (-0.53, 0.41) | 0.815 | 483 | 0.01 | (-0.51, 0.53) | 0.973 |
| **ΔBF%** |  |  |  |  |  |  |  |  |  |  |  |  |
| Crude | 547 | -0.06 | (-0.43, 0.30) | 0.739 | 498 | 0.06 | (-0.32, 0.45) | 0.741 | 462 | 0.04 | (-0.33, 0.41) | 0.831 |
| Adjusted | 547 | 0.07 | (-0.29, 0.43) | 0.706 | 498 | 0.19 | (-0.19, 0.57) | 0.330 | 462 | 0.14 | (-0.22, 0.50) | 0.441 |
| **RMSE (%)** |  |  |  |  |  |  |  |  |  |  |  |  |
| Crude | 550 | 0.05 | (0.01, 0.08) | 0.015 | 534 | 0.05 | (-0.00, 0.10) | 0.071 | 497 | 0.05 | (-0.02, 0.12) | 0.167 |
| Adjusted | 550 | 0.03 | (-0.01, 0.07) | 0.106 | 534 | 0.03 | (-0.03, 0.08) | 0.308 | 497 | 0.03 | (-0.04, 0.10) | 0.443 |
| **NLMD (%)** |  |  |  |  |  |  |  |  |  |  |  |  |
| Crude | 550 | 0.02 | (-0.00, 0.03) | 0.065 | 534 | 0.02 | (-0.00, 0.03) | 0.098 | 497 | 0.02 | (-0.01, 0.04) | 0.166 |
| Adjusted | 550 | 0.01 | (-0.01, 0.03) | 0.283 | 534 | 0.01 | (-0.01, 0.03) | 0.358 | 497 | 0.01 | (-0.01, 0.03) | 0.312 |

^1^ Results presented 6, 12 and 18-month change in outcomes (95% CI) per additional unit higher baseline log hair cortisol

^2^ Abbreviations: Body fat percentage (BF%), body weight (BW), non-linear mean deviation (NLMD), root-mean-square deviation (RMSE).

^3^ Model with exposure, outcome and baseline measure of outcome, only.

^4^ Adjusted for physical activity (steps/day), smoking status, educational level, sex, alcohol, age, height, intervention status, country and initial weight loss.

**Supplementary Table 8.** Association between 12-month concurrent changes in log HCC and body weight, body fat percentage and 12-month body weight variability (participants with less than 10 pg/mg hair excluded).

|  | **n** | **β^1^** | **(95% CI)** | **P** |
| --- | --- | --- | --- | --- |
| **ΔBW^2^ (kg)** |  |  |  |  |
| Crude^3^ | 372 | -0.01 | (-0.63, 0.60) | 0.968 |
| Adjusted^4^ | 372 | -0.17 | (-0.79, 0.46) | 0.600 |
| **ΔBF%** |  |  |  |  |
| Crude | 360 | 0.05 | (-0.44, 0.54) | 0.845 |
| Adjusted | 360 | -0.02 | (-0.50, 0.46) | 0.922 |
| **RMSE (%)** |  |  |  |  |
| Crude | 355 | -0.00 | (-0.08, 0.07) | 0.890 |
| Adjusted | 355 | -0.03 | (-0.10, 0.04) | 0.390 |
| **NLMD (%)** |  |  |  |  |
| Crude | 355 | 0.02 | (-0.01, 0.05) | 0.145 |
| Adjusted | 355 | 0.01 | (-0.02, 0.03) | 0.522 |

^1^ Results presented as change in outcomes (95% CI) between baseline and month 12 per additional unit of 12-month concurrent change in log hair cortisol

^2^ Abbreviations: Body fat percentage (BF%), body weight (BW), non-linear mean deviation (NLMD), root-mean-square deviation (RMSE).

^3^ Model with exposure, outcome and baseline measures of both, only.

^4^ Adjusted for physical activity (steps/day), smoking status, educational level, sex, alcohol, age, height, intervention status, country and initial weight loss.

**Supplementary Table 9.** Association between initial 12-month changes in log HCC and subsequent change in body weight, body fat percentage and variability in body weight between month 12 and month 18 (participants with less than 10 pg/mg hair excluded)

|  | **n** | **β^1^** | **(95% CI)** | **P** |
| --- | --- | --- | --- | --- |
| **ΔBW^2^ (kg)** |  |  |  |  |
| Crude | 341 | 0.09 | (-0.32, 0.49) | 0.675 |
| Adjusted | 341 | -0.13 | (-0.54, 0.27) | 0.512 |
| **ΔBF%** |  |  |  |  |
| Crude | 331 | 0.22 | (0.16, 0.59) | 0.251 |
| Adjusted | 331 | 0.16 | (-0.22, 0.54) | 0.414 |
| **RMSE (%)** | |  |  |  |
| Crude | 302 | 0.03 | (-0.01, 0.08) | 0.173 |
| Adjusted | 302 | 0.02 | (-0.03, 0.07) | 0.359 |
| **NLMD (%)** | |  |  |  |
| Crude | 302 | 0.02 | (0.00, 0.05) | 0.039 |
| Adjusted | 302 | 0.02 | (-0.00, 0.04) | 0.094 |

^1^ Results presented as change in outcomes (95% CI) between month moth 12 and 18 per additional unit change in log hair cortisol between baseline and month 12.

^2^ Abbreviations: Body fat percentage (BF%), body weight (BW), non-linear mean deviation (NLMD), root-mean-square deviation (RMSE).

^3^ Model with exposure, outcome and baseline measures of both, only.

^4^ Adjusted for physical activity (steps/day), smoking status, educational level, sex, alcohol, age, height, intervention status, country and initial weight loss.

**Supplementary Table 10.** Association between baseline log hair cortisol concentration (pg/mg) and subsequent 6-, 12-, and 18-month weight loss maintenance, change in body fat percentage and body weight variability

|  | **Month 6** | | | | **Month 12** | | | | **Month 18** | | | |
| --- | --- | --- | --- | --- | --- | --- | --- | --- | --- | --- | --- | --- |
|  | **n** | **β^1^** | **(95% CI)** | **P** | **n** | **β** | **(95% CI)** | **P** | **n** | **β** | **(95% CI)** | **P** |
| **ΔBW^2^ (kg)** |  |  |  |  |  |  |  |  |  |  |  |  |
| Crude^3^ | 477 | -0.16 | (-0.50, 0.18) | 0.361 | 439 | -0.10 | (-0.56, 0.36) | 0.662 | 410 | 0.22 | (-0.33, 0.77) | 0.435 |
| Adjusted^4^ | 477 | -0.13 | (-0.49, 0.22) | 0.457 | 439 | -0.07 | (-0.55, 0.40) | 0.759 | 410 | 0.19 | (-0.36, 0.75) | 0.492 |
| **ΔBF%** |  |  |  |  |  |  |  |  |  |  |  |  |
| Crude | 467 | -0.11 | (-0.51, 0.29) | 0.586 | 428 | -0.03 | (-0.44, 0.38) | 0.874 | 400 | 0.03 | (-0.39, 0.45) | 0.888 |
| Adjusted | 467 | 0.08 | (-0.31, 0.47) | 0.689 | 428 | 0.21 | (-0.19, 0.61) | 0.299 | 400 | 0.29 | (-0.10, 0.69) | 0.147 |
| **RMSE (%)** |  |  |  |  |  |  |  |  |  |  |  |  |
| Crude | 469 | 0.04 | (-0.01, 0.08) | 0.114 | 455 | 0.05 | (-0.01, 0.11) | 0.126 | 420 | 0.02 | (-0.06, 0.10) | 0.604 |
| Adjusted | 469 | 0.02 | (-0.03, 0.07) | 0.375 | 455 | 0.03 | (-0.03, 0.09) | 0.377 | 420 | 0.00 | (-0.08, 0.08) | 0.970 |
| **NLMD (%)** |  |  |  |  |  |  |  |  |  |  |  |  |
| Crude | 469 | 0.02 | (-0.01, 0.04) | 0.157 | 455 | 0.02 | (-0.00, 0.04) | 0.092 | 420 | 0.01 | (-0.01, 0.04) | 0.294 |
| Adjusted | 469 | 0.01 | (-0.01, 0.03) | 0.438 | 455 | 0.01 | (-0.01, 0.03) | 0.253 | 420 | 0.01 | (-0.01, 0.03) | 0.434 |

^1^ Results presented 6, 12 and 18-month change in outcomes (95% CI) per additional unit higher baseline log hair cortisol

^2^ Abbreviations: Body fat percentage (BF%), body weight (BW), non-linear mean deviation (NLMD), root-mean-square deviation (RMSE).

^3^ Model with exposure, outcome and baseline measure of outcome, only.

^4^ Adjusted for physical activity (steps/day), smoking status, educational level, sex, alcohol, age, height, intervention status, country and initial weight loss.

**Supplementary Table 11.** Association between 12-month concurrent changes in log HCC and body weight, body fat percentage and 12-month body weight variability

|  | **n** | **β^1^** | **(95% CI)** | **P** |
| --- | --- | --- | --- | --- |
| **ΔBW^2^ (kg)** |  |  |  |  |
| Crude^3^ | 380 | 0.41 | (-0.09, 0.92) | 0.111 |
| Adjusted^4^ | 380 | 0.33 | (-0.18, 0.85) | 0.204 |
| **ΔBF%** |  |  |  |  |
| Crude | 374 | 0.23 | (-0.20, 0.67) | 0.295 |
| Adjusted | 374 | 0.15 | (-0.28, 0.58) | 0.487 |
| **RMSE (%)** |  |  |  |  |
| Crude | 368 | -0.01 | (-0.07, 0.05) | 0.770 |
| Adjusted | 368 | -0.02 | (-0.09, 0.04) | 0.470 |
| **NLMD (%)** |  |  |  |  |
| Crude | 368 | 0.01 | (-0.01, 0.03) | 0.234 |
| Adjusted | 368 | 0.01 | (-0.02, 0.03) | 0.635 |

^1^ Results presented as change in outcomes (95% CI) between baseline and month 12 per additional unit of 12-month concurrent change in log hair cortisol

^2^ Abbreviations: Body fat percentage (BF%), body weight (BW), non-linear mean deviation (NLMD), root-mean-square deviation (RMSE).

^3^ Model with exposure, outcome and baseline measures of both, only.

^4^ Adjusted for physical activity (steps/day), smoking status, educational level, sex, alcohol, age, height, intervention status, country and initial weight loss.

**Supplementary Table 12.** Association between initial 12-month changes in log HCC and subsequent change in body weight, body fat percentage and variability in body weight between month 12 and month 18

|  | **n** | **β^1^** | **(95% CI)** | **P** |
| --- | --- | --- | --- | --- |
| **ΔBW^2^ (kg)** |  |  |  |  |
| Crude | 350 | -0.06 | (-0.41, 0.29) | 0.720 |
| Adjusted | 350 | -0.17 | (-0.52, 0.19) | 0.359 |
| **ΔBF%** |  |  |  |  |
| Crude | 346 | 0.29 | (-0.08, 0.65) | 0.124 |
| Adjusted | 346 | 0.23 | (-0.14, 0.60) | 0.221 |
| **RMSE (%)** | |  |  |  |
| Crude | 295 | 0.03 | (-0.01, 0.08) | 0.152 |
| Adjusted | 295 | 0.03 | (-0.02, 0.07) | 0.263 |
| **NLMD (%)** | |  |  |  |
| Crude | 295 | 0.02 | (-0.00, 0.04) | 0.098 |
| Adjusted | 295 | 0.02 | (-0.01, 0.04) | 0.173 |

^1^ Results presented as change in outcomes (95% CI) between month moth 12 and 18 per additional unit change in log hair cortisol between baseline and month 12.

^2^ Abbreviations: Body fat percentage (BF%), body weight (BW), non-linear mean deviation (NLMD), root-mean-square deviation (RMSE).

^3^ Model with exposure, outcome and baseline measures of both, only.

^4^ Adjusted for physical activity (steps/day), smoking status, educational level, sex, alcohol, age, height, intervention status, country and initial weight loss.
